# Supplementary material for: Patterns of Intron Gain and Loss in Fungi
Source: PLoS Biol. 2004 Nov 30;2(12):e422. doi: 10.1371/journal.pbio.0020422 (PMC532390; doi:10.1371/journal.pbio.0020422)
Supplement: Table S1 — Also available at http://genes.mit.edu/NielsenEtAl/. (4.3 MB ZIP). [file pbio.0020422.st001.zip › NielsenEtAl/html/422.html]

AN7487.1.NCU06454.1.MG00466.1.FG05447.1


```
 CLUSTAL W (1.82) Multiple Sequence Alignments - Introns Inserted


Sequence 1: MG00466.1	194 aa
Sequence 2: FG05447.1	195 aa
Sequence 3: AN7487.1	192 aa
Sequence 4: NCU06454.1	207 aa
Alignment Length: 208 aa
Number Identitical Residues: 147 aa
Alignment Score (without introns) 6295


MG00466.1 	-MVVATIK2-----------~CVVVGDGAVGKTCLLISYTTNKFPSEYVPTVFDNYAVTV
NCU06454.1	-MVTGTIK~YAKTNHPLSRE2CVVVGDGAVGKTCLLISYTTNKFPSEYVPTVFDNYAVTV
FG05447.1 	MAVVATIK2-----------~CVVVGDGAVGKTCLLISYTTNKFPSEYVPTVFDNYAVTV
AN7487.1  	-MVVATIK2-----------~CVVVGDGAVGKTCLLISYTTNKFPSEYVPTVFDNYAVTV
          	  *..***             ***************************************

MG00466.1 	M2IGDEPYTLGLFDTAGQEDYDRLRPLSYPQTDVFLVCFSVTSPASFENVREKWFPEVHH
NCU06454.1	M2IGDEPYTLGLFDTAGQEDYDRLRPLSYPQTDVFLICFSVASPASFENVSQKWAPEVNH
FG05447.1 	M2IGDEPYTLGLFDTAGQEDYDRLRPLSYPQTDVFLVCFSVTSPASFENVREKWFPEVRH
AN7487.1  	M2IGDEPYTLGLFDTAGQEDYDRLRPLSYPQTDVFLVCFSVTSPASFENVREKWFPEVHH
          	* **********************************:****:******** :** ***.*

MG00466.1 	HCPGVPCLIVGTQVDLRDDPSVREKLSKQKMQPVRREDGERMAKELGAVKYVECSALTQY
NCU06454.1	HCPGVPFLIVGTQKDLRSDKELRDKLAQRKQSMIEFKQGEKLAQDLDAVKYVECSALTQE
FG05447.1 	HCPGVPCLIVGTQVDLRDDPSVREKLSKQKMQPVRREDGERMAKDLGAVKYVECSALTQY
AN7487.1  	HCPGVPCLIVGTQVDLRDDPAVRDKLARQKMQPIRKEDGDRMAKDLGAVKYVECSALTQY
          	****** ****** ***.*  :*:**:::* . :. ::*:::*::*.************ 

MG00466.1 	KLKDVFDE0AIVAALEPP--TPKRKSKKCLIL
NCU06454.1	GLKNVFDE0AIVAALEPPQKKTSKRDKKCLIL
FG05447.1 	KLKDVFDE0AIVAALEPP--APKKKSHKCLVL
AN7487.1  	KLKDVFDE0AIVAALEP---APKKRS-RCVLL
          	 **:**** ********    ..::. :*::*
```
